# Buchanan Redivivus

**Published:** 1880-10

**Authors:** 


BUCHANAN REDIVIVUS, THE BOGUS DIPLOMA VENDER is again in the flesh, and safely lodged in jail in Philadelphia. Should this hoary headed and notoriously unprincipled scoundrel ever succeed in outwitting the law, his former villianies will doubtless be renewed in the new quarters in which he may take up his abode. His agents already know his whereabout and his wishes, and his *stock in trade* will doubtless soon be scattered in those localities where they command the readiest sale. But seriously, this old rascal compels some attention, though he has our utter contempt, by the comparative *boldness* of his operations, while some of the other "diploma mills," attempt through cunning and duplicity, to lay some claim to respectability. Buchanan's very boldness, places him a degree or two *higher* in the moral scale, than that of such "professors" as deal out "sheepskins" in a more popular, if not respectable manner.

Some of these "professors," like their great prototype in the business, have been dragged from their obscurity only by the advertisement of themselves in circulars, and being allowed to append their little names to the diplomas of the schools to which they belong. But enough of this nefarious business for the present.

